# Methods for the Preparation of Large Quantities of Complex Single-Stranded Oligonucleotide Libraries

**DOI:** 10.1371/journal.pone.0094752

**Published:** 2014-04-14

**Authors:** Yusuf E. Murgha, Jean-Marie Rouillard, Erdogan Gulari

**Affiliations:** 1 Department of Biomedical Engineering, University of Michigan, Ann Arbor, Michigan, United States of America; 2 Department of Chemical Engineering, University of Michigan, Ann Arbor, Michigan, United States of America; University of Helsinki, Finland

## Abstract

Custom-defined oligonucleotide collections have a broad range of applications in fields of synthetic biology, targeted sequencing, and cytogenetics. Also, they are used to encode information for technologies like RNA interference, protein engineering and DNA-encoded libraries. High-throughput parallel DNA synthesis technologies developed for the manufacture of DNA microarrays can produce libraries of large numbers of different oligonucleotides, but in very limited amounts. Here, we compare three approaches to prepare large quantities of single-stranded oligonucleotide libraries derived from microarray synthesized collections. The first approach, alkaline melting of double-stranded PCR amplified libraries with a biotinylated strand captured on streptavidin coated magnetic beads results in little or no non-biotinylated ssDNA. The second method wherein the phosphorylated strand of PCR amplified libraries is nucleolyticaly hydrolyzed is recommended when small amounts of libraries are needed. The third method combining *in vitro* transcription of PCR amplified libraries to reverse transcription of the RNA product into single-stranded cDNA is our recommended method to produce large amounts of oligonucleotide libraries. Finally, we propose a method to remove any primer binding sequences introduced during library amplification.

## Introduction

The last decade has seen the emergence of a broad range of applications for microarray-based DNA and RNA oligonucleotide libraries. In synthetic biology, DNA oligonucleotides are the building blocks for the assembly of single genes [1]–[3] to whole genomes [4], [5]. Targeted next-generation sequencing relies heavily on oligonucleotide libraries as a source of baits to capture, either in the form of DNA padlock probes for the circularization of targeted sequences [6], [7] or in the form of RNA baits for the direct capture of sequencing genomic DNA library fragments (Agilent's SureSelect, MYcroarray's MYbaits [8]). Millykangas et al. pushed the application of oligonucleotide libraries for targeted sequencing even further by integrating the target capture into the sequencing device, using a DNA oligonucleotide library to customize the primer lawn on a sequencing flowcell [9]. Similarly, oligonucleotide libraries are used for sequence-specific priming of molecular reactions such as reverse transcription. Oligonucleotides libraries are also widely used to encode active RNA such as shRNA [10], [11], or peptides [12], [13] after cloning in appropriate vectors. Recently, fluorescently labeled oligonucleotide libraries as molecular detection probes in fluorescent in situ hybridization (FISH) techniques such as OligoPaint [14].

While it is technically possible to separately synthesize each oligonucleotide of a library in a column, this process becomes cost prohibitive as the number of sequences increases. Synthesis prices can be greatly reduced by using massively parallel synthesis technologies primarily developed for manufacturing DNA microarrays. This has been achieved by using various methods including photodeprotection (Affymetrix) electrochemical acid generation (Combimatrix), inkjet printing of synthesis reagents (Agilent) and photo-generated acid deprotection (MYcroarray). However, the major drawback of all massively parallel DNA synthesis technologies is the relatively small amount of oligonucleotides (femtomole scale of each sequence) produced on planar substrates. The yield of synthesized oligonucleotides released from a microarray can be increased by an initial PCR amplification step. This leads to the formation of double-stranded DNA (dsDNA) flanked by PCR primer sequences, hence the need for a robust procedure to remove the complementary strand and both primer sequences.

Current methods to convert double-stranded (dsDNA) to single-stranded (ssDNA) can be broadly divided into two categories, enzyme-based and affinity selection-based. The enzymatic methods either selectively hydrolyze the undesired strand or preferentially amplify the desired strand. Three enzymes that hydrolyze duplex DNA are exonuclease III, T7 exonuclease and lambda exonuclease. Of these, exonuclease III and T7gp6 exonuclease digest both strands of dsDNA at equal rates to form two shorter DNA fragments of half the dsDNA template length [15], [16]. Thus to prepare full length ssDNA, the desired strand is modified to confer nuclease resistance. As exonuclease III hydrolyzes dsDNA in 3′->5′ directions and is not active on ssDNA, the PCR product is digested with type II restriction enzymes to generate four-base or longer 3′-protrusions. This enzyme has been used to prepare templates for chain terminator sequencing [17]. In contrast, T7 exonuclease cannot hydrolyze strands with 4 or more phosphorothioate bonds at 5′-end nucleic acid backbone. Here, phosphorothioate primers are used to prepare single-strand templates for single nucleotide polymorphisms (SNP) detection assays and sequencing [18]. Lastly, lambda exonuclease preferentially hydrolyzes the 5′ terminus phosphoryl strand of duplex DNA, while it displays greatly reduced activity for 5′ terminus hydroxyl strand of native and denatured DNA [19], [20]. Also, the enzyme does not cleave 5′- modified termini (digoxigenin, biotin and cyanine dyes) and has been used to prepare single-stranded DNA for hybridization assays [21]–[23], sequencing [24], [25] and systematic selection of aptamers (SELEX, Systematic Evolution of Ligands by EXponential enrichment) [26].

Alternatively, the preferential amplification of one strand is achieved by using unequal primer concentrations during PCR (asymmetric PCR). Once the limiting primer is exhausted, the excess primer extends to form ssDNA during the rest of the cycles. This method requires rigorous primer design [27] and although more than 50+ cycles are required to produce high ssDNA to dsDNA ratio, variations of asymmetric PCR have been adopted for microarray and pyrosequencing applications [28]–[31]. Finally, affinity selection method is based on immobilization of one of the amplicons strands to a solid-support. Here the biotinylated strand remains bound to streptavidin conjugated paramagnetic beads, while the non-biotinylated strand is released into the solution when heated (> melting temperature) or made alkaline (> pH 12.1) [32]–[34].

In the methods described above, the primer binding sequences (PBS) used to prime the PCR amplification are not removed. This is a major issue for applications that require PBS-free oligonucleotide libraries. Nicking endonucleases have been used to prepare PBS-free ssDNA from PCR amplified microarray synthesized oligonucleotide libraries. The PBS-free desired strand is melted from complementary strand and purified on denaturing polyacrylamide gel [35]. Gel purification results in >70% loss of product and is not feasible to prepare oligonucleotide libraries containing variable template lengths. In this paper, we describe three procedures to get high quality, full length PBS-free ssDNA oligonucleotide libraries by affinity selection starting from microarray-synthesized oligonucleotide. The first two methods are adaptations of existing methods namely, alkaline denaturation of non-bead bound strand and lambda exonuclease hydrolysis of undesired DNA strand. The third procedure is a novel application of two frequently used molecular biology techniques, transcription and reverse transcription. The enzyme reverse transcriptase in the presence of a suitable primer synthesizes a complementary DNA (cDNA) copy of an RNA template [36], [37]. This combined with an upstream RNA amplification of short synthetic DNA templates (<150 bp) has the potential to produce large amount of ssDNA [38]. The methods can be tailored to remove one or both primer binding sequences (PBS). The experimental design and removal of PBS is explained in the results section.

## Materials and Methods

### Reagents

Restriction enzymes (BspQI, Nt.BspQI, Nb.BtsI), lambda exonuclease and hot-start Phusion PCR polymerase are obtained from New England Biolabs. DNA exonuclease I and Antartic phosphatase are obtained from Fermentas. All enzymes are used per manufacturer's recommendation unless otherwise noted. PCR primers are obtained from IDT. Primer sequences are described in Table 1. Nucleotide removal, PCR purification, MinElute and RNeasy kits are obtained from Qiagen. Size-exclusion spin columns (CentriSpin20) are obtained from Princeton Separation. The streptavidin-coated magnetic beads used in this work are the MyOne Streptavidin C1 Dynabeads obtained from Invitrogen. Fresh alkaline melting solution is prepared by adding 125 µl of 10N sodium hydroxide (72068, Sigma-Aldrich) to 9.875 ml of molecular grade water. This solution should be discarded at the end of the day. 1N Hydrochloric acid is obtained from (H9892, Sigma-Aldrich. Reagents for emulsion polymerase chain reaction bovine albumin serum (B8667), Triton X100 (T8787), mineral oil (M5904) and diethyl ether (32203) are obtained from Sigma-Aldrich and ethyl acetate (E145) from Fisher Scientific. Finally, the surfactant ABIL EM90 is obtained from Evonik. DNA microarrays were obtained from MYcroarray (Ann Arbor, MI)

**Table 1:** Primer sequences.

Name	Sequence (5′ – 3′)*
P1	Biotin-GGAGGTAGTATGGCAGTG
P2	Biotin-CCTATCCATCGCTCTTCG
P3	P-GAATTGTAATACGACTCACTATAGGGAGAATGCACGCAGTG
P4	Biotin-TAATACGACTCACTATAGGGAGACGCAAGTCAGCTCTTCG
P5	TATAGGGAGACGCAAGTCAGCTCTTCG
P6	GGGAGAATGCACGCAGTGNN
P7	CGAAGAGCTGACTTGCGTCT
P8	Biotin-(dA)_30_ GGGAGAATGCACGCAGTGNN
P9**	GGAGGCCGGAGAATTGTAATACGACTCACTATAGGGAGAGGGCTCTTCATGG
P10	CTGACCTTAAACCTAACGCGAGGGCGGCAGTTGGGATTTCGTGACCTATGCAGCTCTTCG
P11	Biotin-GCTGACCTTAAACCTAACGCGAGGGCGGCAGTTGGGATTTCGTGACCTATGCAGCTCTTC

*The restriction enzyme sequence is underlined.

**T7 promoter sequence is in bold.

### Oligonucleotide library and Emulsion PCR

Custom oligonucleotide libraries (MYlib) are obtained from MYcroarray (Ann Arbor, MI). They are amplified as is using optimized emulsion PCR protocol (Y. Murgha, in preparation) adapted from Williams et al [39]. For a typical reaction, the PCR mixture or aqueous phase (100 µl) consists of 2.5 femtomoles of template oligonucleotide library, 0.5 µM each of forward and reverse universal primers, 0.2 mM dNTPs, 0.5 µg/µl bovine serum albumin, 4 units of Phusion Hot-Start polymerase and 1x GC rich buffer (contains 1.5 mM MgCl_2_). The oil phase consists of 4% ABIL EM90 and 0.05% Triton X 100 in mineral oil. The oil phase is continuously stirred at 1000 rpm at 4°C and the aqueous phase is added drop-wise. The forming emulsion is stirred for an additional 15 minutes at 1000 rpm (4°C). Once the emulsion is formed, the reaction is incubated at 98°C for 2 min to activate Phusion polymerase followed by 30 cycles of 15 sec at 98°C, 25 sec at primer Tm - 2°C, and 25 sec at 72°C and a final extension at 72°C for 5 min in a (Mastercycler EP gradient) thermocycler (Eppendorf).

The emulsion is broken by successive washes with 1 ml water-saturated diethyl ether and ethyl acetate (fume hood). The final wash is done with diethyl ether. Any remaining diethyl ether in the tubes is evaporated by incubation at 37°C (10–15 min). The amplicons of each 100 µl reaction is purified on silica based spin columns (Qiaquick PCR purification kit) following manufacturer's protocol with an additional washing step to completely remove salts.

### Alkaline Denaturation

An emulsion PCR is performed with two biotinylated PCR primers (Table 1: primers P1, P2) such that both strands of the amplicon have 5′-biotin. In this work, the primer sequences at the 5′ and 3′ end of antisense strand have recognition site for nicking endonuclease Nt.BspQI (5′—GCTTCCTN|—3′) and Nb.BtsI (5′—NN|CACTGC—3′). The emulsion PCR product (25 pmol; ∼1.8 µg) is digested with 60 U of Nb.BtsI in 80 µl reaction at 37°C for 2 h, followed by purification on PCR MinElute column. The cleaned product is digested with 30 U of Nt.BspQI in 50 µl reaction at 50°C for 2 h, followed by enzyme heat inactivation at 80°C for 20 min. The products are bound to 50 µl of streptavidin coated paramagnetic beads following manufacturer's protocol with following modifications except that the binding is performed for 30 min on rotator at room temperature. The antisense strand is denatured from its complementary bead bound sense strand by two successive 2 min room temperature incubations with 40 µl 0.125 M sodium hydroxide (freshly prepared). The supernatant of both sodium hydroxide washes is saved and combined, which is neutralized with 12 µl 1 M HCl (0.125 M HCl) and 8 µl 1 M Tris-HCl pH 8 (100 mM). Once precipitated (add 5 µg linear acrylamide), 10 µl DNA is hybridized with excess P1-coated magnetic beads (∼1.2–1.5-fold) for 30 min at room temperature. The unbound ssDNA product is cleaned on centrispin20 column.

### Lambda Exonuclease

An emulsion PCR is performed with modified primers (Table 1: primers P3 and P5) such that the desired strand is biotinylated at its 5′end and the unwanted complementary strand is phosphorylated at its 5′end. Lambda exonuclease is used to hydrolyze the 5′-phosphoryl strand of double-stranded DNA. In a typical reaction, 1–2 µM amplicons are incubated at 37°C for 60 min with 1 U enzyme for every 5 pmol dsDNA. The enzyme is inactivated by heating at 70°C for 15 min. The single-stranded DNA is cleaned up on centrispin 20 size exclusion column.

### *In Vitro* Transcription and Reverse Transcription

4 pmol emulsion PCR product is transcribed at 42°C for 4 h (40 µl) with AmpliScribe™ T7-flash RNA amplification kit (ASF3257,Epicentre Biotechnologies). The template DNA is digested with 2 U of TURBO DNase for 15 min at 37°C, followed by 5 min room temperature enzyme inactivation with 3 µl of DNase inactivation reagent (AM1907, Ambion Inc.). The DNase-treated products are purified on two separate RNeasy isolation columns and eluted with 50 µl of nuclease-free water. In a typical RT reaction, 100 picomoles of RNA is reverse transcribed with 1.5-fold excess biotinylated primer (150 pmol) in solution containing 50 mM Tris-HCl (pH 8.3), 75 mM KCl, 3 mM MgCl_2_, 10 mM dithiothreitol, 1 mM deoxynucleotide triphosphates, 10 units of SUPERase-In RNase inhibitor (Ambion Inc.) and 200 units of superscript II (Invitrogen) per 20 µl volume. This reaction is linearly scaled-up to process 1200 pmol RNA. The RT reaction is incubated at 37°C for 3 h, followed by brief denaturation of RNA:cDNA at 95°C for 5 min and immediate cooling on ice (2 min). The RNA is hydrolyzed with 0.43 volumes 1 M NaOH (0.3 M) at 65°C for 15 min, and then neutralized with equal amount of 1 M HCl. The resultant biotinylated cDNA is precipitated with 10 µg linear acrylamide (AM9520, Ambion Inc.), 0.3 M sodium acetate and 2.5 volumes 100% ethanol. The cDNA pellet is suspended in 100 µl nuclease free water. Finally, cDNA is purified on four separate centrispin 20 columns (25 µl each) to remove excess nucleotides.

### Removal of universal PCR primer sequences and Affinity purification

#### **Hybridization**

The biotinylated cDNA (approx. 100 pmol or 3.5 µg) is hybridized with 2.5-foldexcess Nb.BtsI forward restriction oligo (P6) and 1.5-fold BspQI reverse restriction oligo (P7). The hybridization is done in 50 mM potassium acetate, 20 mM Tris-acetate, 10 mM magnesium acetate and 1 mM dithiothreitol (1x NEB buffer 4; New England biolabs) solution supplemented with 1x bovine albumin serum. The solution is incubated at 80°C for 2 min to melt DNA strands, followed by slow cooling (−0.1°C/s) to 37°C (50 ul)

#### **Sequential removal of PCR primer ends with on-bead BspQI digestion**

Post hybridization, the 3′-end PCR primer containing BtsI recognition site is digested with 50 U Nb.BtsI at 37°C for 2 h (total reaction volume 100 µl; 1x NEB, 1x BSA) followed by capture of cDNA on streptavidin coated magnetic beads. 50 µl of beads (250 pmol binding capacity) is used to immobilize 5′-biotinylated cDNA. Once washed the beads are suspended in 100 µl binding solution (2 M NaCl, 1 mM EDTA, 10 mM Tris-HCl pH 7.5) to which is added 100 µl of the Nb.BtsI digestion reaction and incubated at room temperature for 15 min with gentle shaking. The biotinylated cDNA coated beads are collected with magnetic stand and unbound material (i.e. BtsI supernatant) is aspirated out and saved for further digestion. The beads are washed twice with 200 µl 1x NEB buffer 4, followed by suspension of beads in 100 µl of enzyme mix containing 50 U BspQI enzyme, 1x NEB buffer 4 and 1x BSA. The reaction is done at 50°C for 30 min and put on ice for 1–2 min. The beads are collected on magnetic stand and the supernatant (contains ssDNA probes) is aspirated out and saved for precipitation. The beads are regenerated and reused for one round of negative selection to capture BspQI uncut biotinylated cDNA and BtsI uncut cDNA. The regenerated beads are stored in 50 µl binding solution. Immediately after precipitation the biotinylated cDNA is hybridized in 50 µl with 0.5-fold excess biotinylated Nb.BtsI forward restriction oligo (P8) prior to negative selection on beads.

### Bead regeneration

The bead regeneration protocol is adapted from [40]. The biotinylated DNA coated beads are suspended in nuclease free water (5 µg/µl) and incubated at 70°C for 30 s, followed by immediately cooling on ice for 2 min to release the biotinylated DNA strand from streptavidin coated beads. The beads are collected on tube wall with magnetic stand and the solution is aspirated out and discarded. The beads are washed once with equal volume of water and finally suspended in binding buffer (2 M NaCl, 1 mM EDTA, 10 mM Tris-HCl pH 7.5) for reuse.

### Determination of the single strand nature of the DNA by exonucleolytic hydrolysis

An aliquot (100 nanograms) of ssDNA (cDNA) formed is digested with 20 U of single-stranded DNA specific exonuclease I and 1 U of antarctic phosphatase for 15 min at 45°C (10 µl). 50 mM EDTA (pH 8.0) is added to the reaction and the enzyme is heat inactivated by incubation at 80°C for 20 min.

### Determination of library complexity

#### Generation of biotinylated cDNA targets

Biotinylated cDNA is produced from microarray oligonucleotide library by emulsion PCR, *in vitro* transcription and reverse transcription (described above) with few modifications. First, the emulsion PCR is performed with primers P9 and P10 (Table 1). Second, the transcription reaction is scaled down (20 µl) for 2 pmol (∼200 ng) template input. Third, TURBO DNase is heat inactivated by adding 1 µl 0.5 M EDTA pH 8.0 (25 mM) followed by incubation at 65°C for 20 min. Fourth, The DNase-treated products are purified on an RNeasy column and eluted twice with 50 µl of nuclease-free water. Fifth, 1000 pmol (∼40 µg) of RNA elute is reverse transcribed with 1.2-fold excess biotinylated primer (P11, Table 1) and finally the biotinylated cDNA (80 µl) is purified on 2 separate centrispin 20 columns (40 µl each).

#### Microarray processing

Biotinylated cDNA is hybridized overnight (45°C for 18 h) to complementary probe (46–47mer) array in single gasket Agilent cassette (G2534A). The hybridization solution (600 µl) contains 6x SSPE, 0.01 µg µl^−1^ acetylated BSA, 0.05% Tween 20, 5% formamide and 15 µg biotinylated cDNA targets. Prior to loading, the solution is denatured at 65°C for 5 min and cooled on ice for 2 min. Post hybridization, the gasket is removed from array slide under 1x SSPE, washed twice for 3 min with 1x SSPE and once with 0.25x SSPE solution (under gentle stirring). Next, the array slide is immersed in 30 ml solution containing 4 µg Streptavidin Alexa Fluor 555 conjugate (S32355, Invitrogen), 6x SSPE, 0.1 µg µl^−1^ acetylated BSA, 0.05% Tween 20 and incubated for 2 h at 16°C. The slide is washed with 1x SSPE followed by 0.25x SSPE (3 min each), dried (spin 10 s) and scanned with Axon GenePix 4000B (multiple 532 PMT settings).

#### Microarray data analysis

The median spot signal intensity is sorted according to probe ID (6 probes per cDNA molecule). Next, probes with coefficient of variance (CV) >0.55 pass spot quality filter. Of these, cDNA with 2 or more CV passed probes have calculated their trimmed mean intensity (discard highest and lowest value). All cDNA probes with intensity >3000 (∼3.5–5 x background; background  = 850 and 630) are denoted as present and rest as absent.

## Results

### Alkaline denaturation

**Figure 1:**
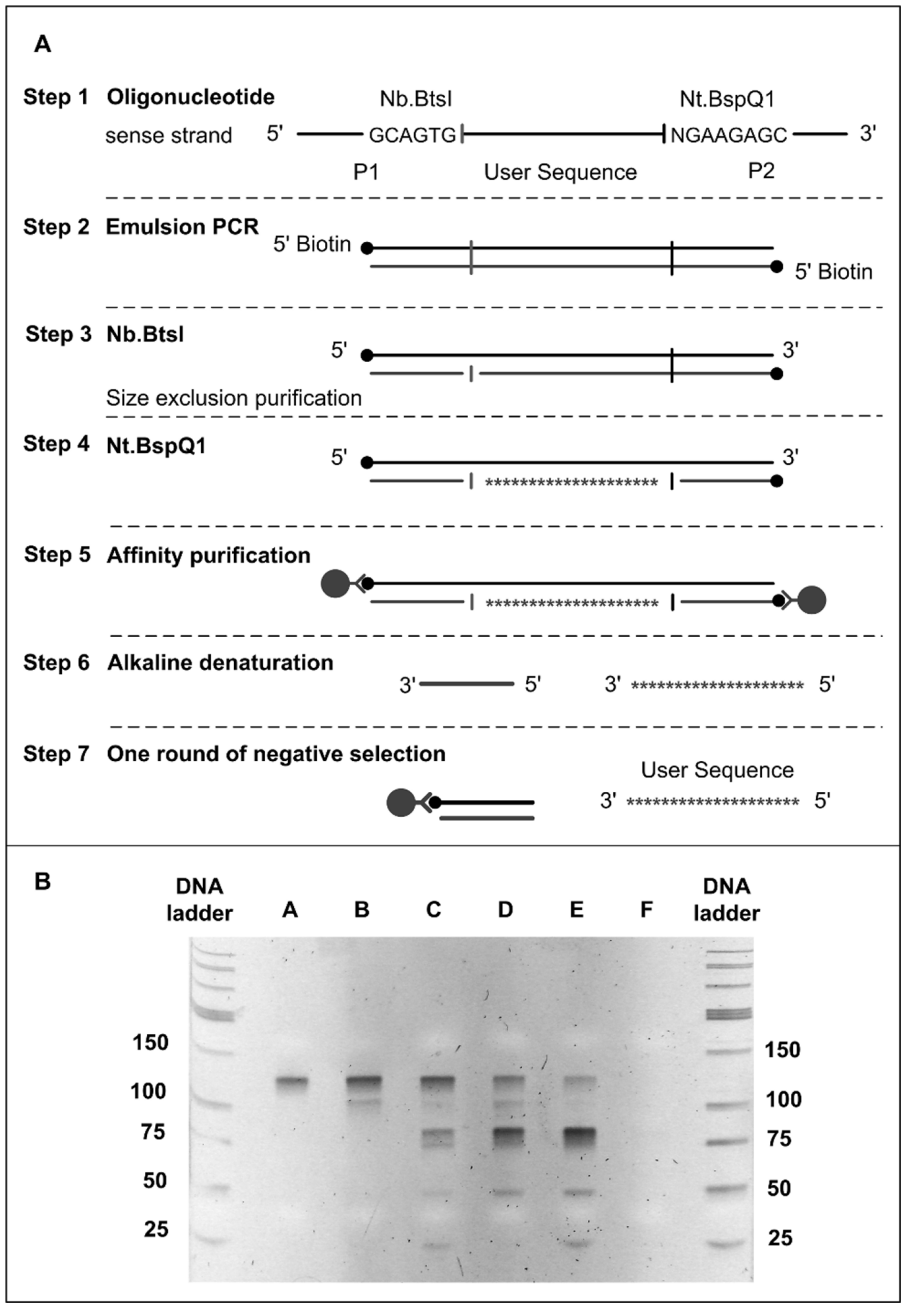
Alkaline denaturation method. (A) Experimental design. (B) lane A – emulsion PCR product (step2), lane B – Nb.BtsI nicked ssDNA (step 3), lane C – nicked DNA (step 4), lane D alkali-melting of nicked ssDNA (step 6), lane E – one of negative selection (step 7), lane F – exonuclease I hydrolysis step 7 products.

The experimental design is presented in Fig 1a. An oligonucleotide library (containing approximately 16,000 sequences in the present case (step 1) is amplified by emulsion PCR using biotinylated universal PCR-primers for immobilization onto streptavidin-coated magnetic beads (step 2). The primer (P1; table 1) present at 5′-end of sense strand has an Nb.BtsI recognition sequence (5′—GCAGTG—3′) and the primer (P2; table 1) at 3′ end has an Nt.BspQI recognition sequence (5′—NGAAGAGC—3′). The endonuclease recognition sites are orientated to nick and remove the priming sequences from the desired strand only. After sequential digestion with both nicking endonucleases (step 3 and 4), the nicked PCR products are bound to streptavidin coated magnetic beads (step 5). Sodium hydroxide is used to melt the non-bead bound DNA strand (step 6). The elute products are neutralized and processed on P1-coated magnetic beads to capture and remove complementary primer fragments released during alkali-melting (step 7). The negative selection elute is purified on size-exclusion column to get ssDNA free of PCR-primer binding sequences (PBS).

The products are run on denaturing polyacrylamide gel [Fig 1b]. The emulsion PCR amplicons (118 bp) are observed at the expected size (Lane A). The primer binding sites (P1, P2) on desired strand of dsDNA are sequentially nicked with Nb.BtsI (Lane B) and Nt.BspQI (Lane C) to yield denatured DNA strands of size 118 bp (PBS-intact; Lane B - E), 100 bp (one PBS intact; Lane B - E), 82 bp (PBS-free; Lane C - E) and 18 bp primers (Lane C, E; not visible in Lane B, D). The unknown band at 50 bp ladder mark (Lane C – E), may be biotinylated primers. Lane D shows the DNA products obtained after alkali-melting. Besides expected DNA bands (82 bp, 18 bp), there are additional bands (118 bp, 100 bp uncut BspQI) that correspond to biotinylated DNA (also seen in Lane E). This may be due breakage of biotin-streptavidin bond under mild alkaline conditions (0.15 M NaOH, 2 min). Attempts to remove released complementary P1 primer by selection on P1-coated beads gives in-sufficient capture on beads (18bp, Lane E). The formation of ssDNA is confirmed by hydrolysis with single-strand exonuclease (Lane F).

### Exonucleolytic hydrolysis of 5′-phosphorylated DNA strand

**Figure 2:**
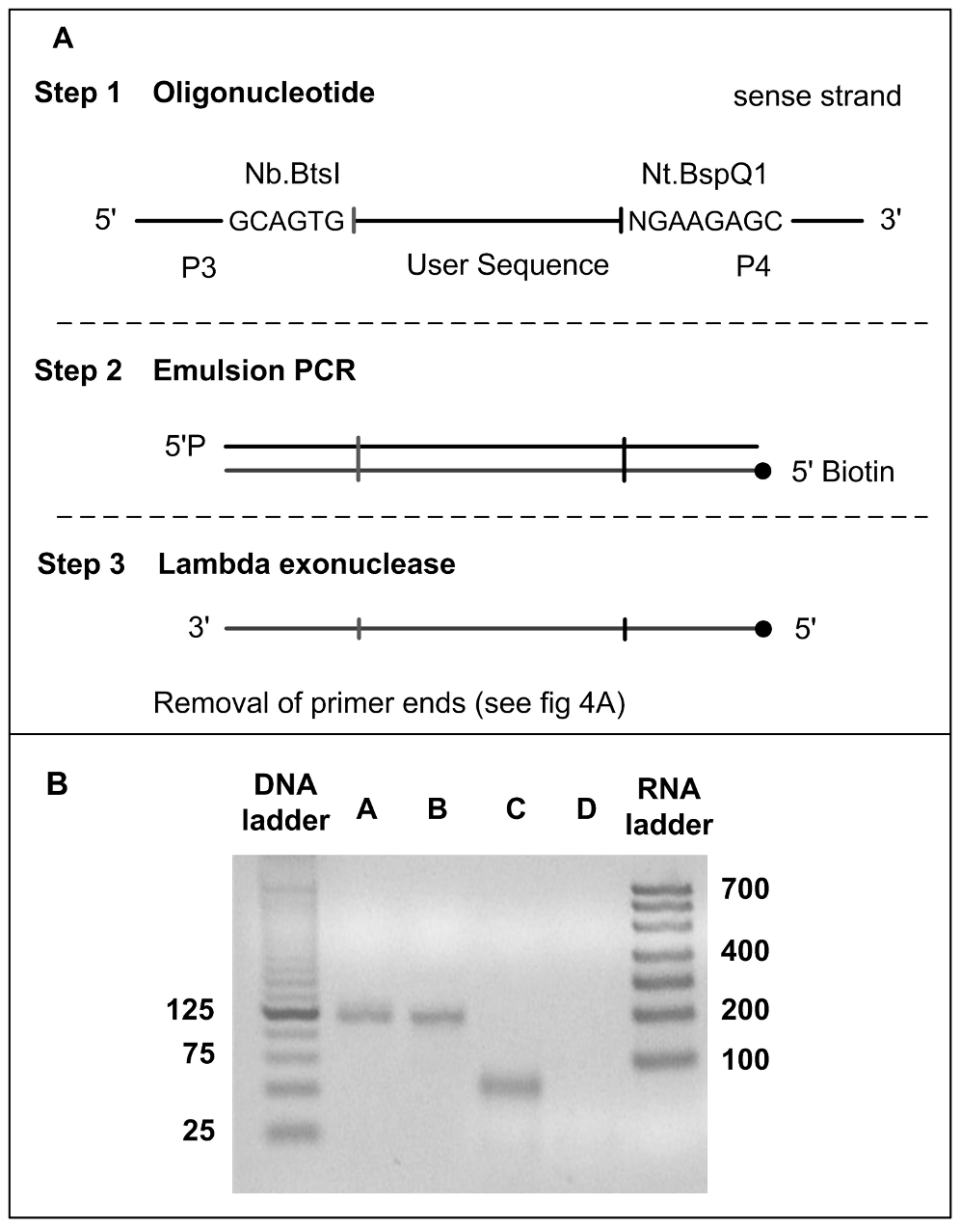
Exonucleolytic hydrolysis of 5**′**-phosphorylated strand. (A) Experimental design. (B) lane A – emulsion PCR product (step 2), lane B – exonuclease I hydrolysis of PCR product, lane C – ssDNA product of the lambda exonuclease treatment after removal of PBS (fig. 4 step 6; c), and lane D – exonuclease I hydrolysis of PBS-free ssDNA.

The experimental design to prepare single-stranded DNA minus PBS is shown in Fig 2a. A chip-derived DNA library is amplified by emulsion PCR to give one strand with 5′-terminus phosphoryl group and complementary strand 5′-terminus biotin group (step 2). The primer (P3; table 1) present at 5′-end of sense strand has an Nb.BtsI recognition sequence (5′—GCAGTG—3′) and the primer (P4; table 1) has an Nt.BspQI recognition site (5′—NGAAGAGC—3′) at 3′ end. The phosphorylated strand is hydrolyzed by enzyme lambda exonuclease while the complementary strand is protected by 5′ termini biotin moiety (step 3). The PCR-primer ends of desired biotinylated strand can be removed by methods presented below (Removal of universal PCR primer sequences).

A library of 4852 oligonucleotides (122 to 124mer, for a desired length of 49 – 51mer after removal of the PBS) is amplified by emulsion PCR using primers P3 and P4 (Table 1) for a final amplicon size of 130–132 bp. The amplicons are observed at the expected size on an agarose gel (Figure 2b, lane A). The 5′ phosphoryl strand is hydrolyzed to produce biotinylated ssDNA. Lane C shows the biotinylated ssDNA after the removal of PBS (49 – 51 mer). The formation of ssDNA is confirmed by digestion of both DNA types with single-strand specific exonuclease. As expected, exonuclease I does not degrade duplex DNA (lane B) but efficiently hydrolyzes ssDNA (lane D). Finally, the amount of product obtained from 100 µl emulsion PCR is on an average 25 pmol (∼1.8 µg), which yields 20 pmol (∼0.72 µg) biotinylated ssDNA with primer binding sequences (PBS).

### *In vitro* transcription and reverse transcription

**Figure 3:**
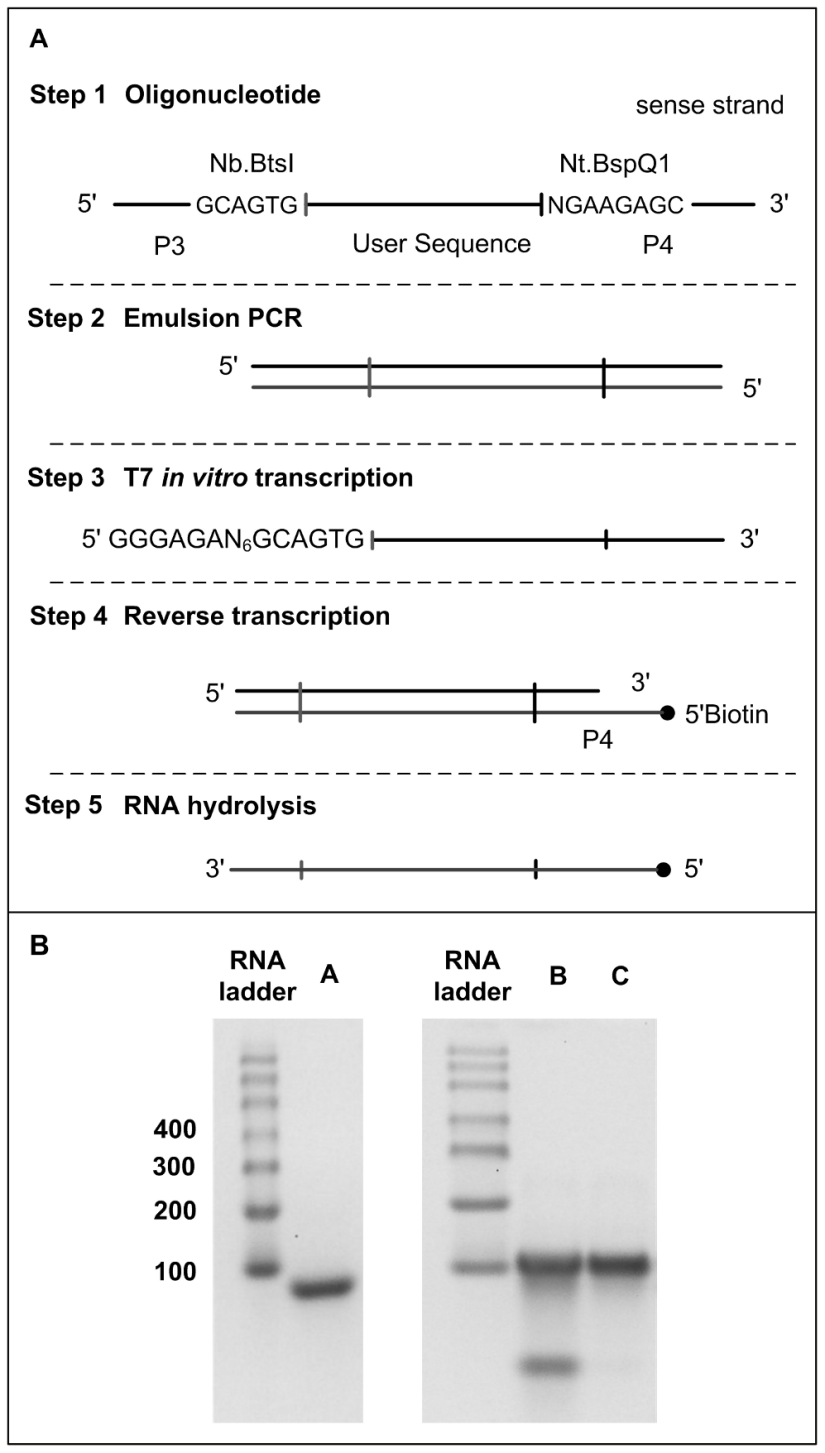
*In vitro* transcription and reverse transcription (IVT-RT). (A) Experimental design. (B) 2% agarose gel electrophoresis. An RNA ladder is used as single-stranded ladder. lane A – RNA (step 3), lane B – cDNA (step 5), and lane C – spin-column purified cDNA (step 5).

**Figure 4:**
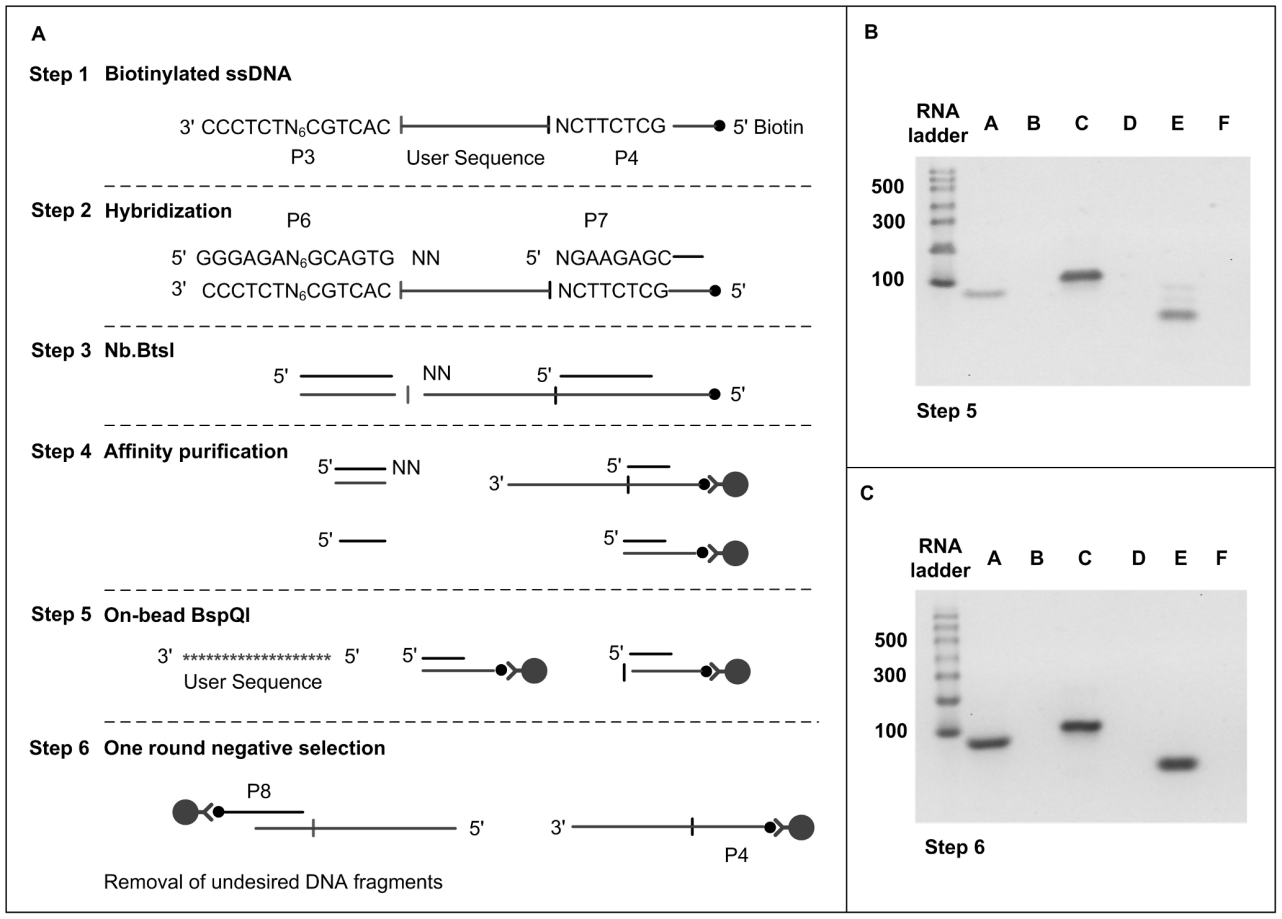
Removal of universal PCR primer sequences. (A) Experimental design. (B,C) 2% agarose gel electrophoresis. An RNA ladder is used as single-stranded ladder. lane A – RNA, lane B – sodium hydroxide degradation of RNA, lane C – cDNA (step 1), lane D – exonuclease I hydrolysis of cDNA, lane E – 50 bp ssDNA (step 5; b), and lane E – 50 bp ssDNA (step 6; c), and lane F – exonuclease I hydrolysis of ssDNA.

The experimental design is shown in Fig 3a. The oligonucleotide library above is amplified by emulsion PCR (step 2) with primers P5 and P6 (Table 1). The primer on the strand complementary to final single-strand DNA product, P5 in this case, has T7 RNA polymerase promoter sequence for *in vitro* transcription. The library of PCR amplicons is used as template in an in vitro transcription reaction (step 3). The resultant RNA library is primed with biotinylated sequence-specific primer (P6; table 1) to make cDNA copies (step 4). Following hydrolysis of the RNA strand with sodium hydroxide, we obtain the desired ssDNA library (step 5). The ssDNA have at their 5′ end BspQI (5′—GCTCTTCN|—3′) and at their 3′ end Nb.BtsI (5′—|CACTGC—3′) recognition sites for removal of primer sequences by methods shown in figure 4.

A library of 4852 oligonucleotides (122 to 124mer, for a desired length of 49 – 51mer after removal of the PBSs) is amplified by emulsion PCR using primers P5 and P6 (Table 1) for a final amplicons size of 117–119 bp (data not shown). Figure 3b shows the products of *in vitro* transcription – reverse transcription (IVT-RT). T7 RNA polymerase transcription of emulsion PCR product gives RNA band of the expected size (94–96 bases, lane A). Lane B shows the cDNA library made by reverse transcription after degradation of RNA at the expected size (107–109 nt). The gain in length is due to the presence of extra sequences at the 5′end of the reverse transcription primer. The low molecular weight DNA band observed in lane B corresponds to the reverse transcription primer. This product is purified with silica-based spin column (PCR Qiaquick purification kit) to remove nucleotides, which results in removal of excess reverse transcription primer (lane C). Starting from 4 pmol PCR amplicons, we obtain on average 3000 pmol spin-column purified RNA, which following reverse transcription results in 1500 pmol biotinylated cDNA. This translates to 7500 pmol of biotinylated cDNA from a 100 µl emulsion PCR (i.e. 20 pmol amplicons gives 15000 pmol RNA). This equates to a 3.75 ml emulsion PCR reaction to make equivalent amount of biotinylated ssDNA with both PBS.

### Removal of universal PCR primer sequences

The experimental design to remove PBS and purify ssDNA with streptavidin-coated magnetic beads is presented in Fig 4a. The 5′-biotinylated ssDNA libraries formed by lambda exonuclease or IVT-RT methods have restriction sites to remove PBS (step 1). Oligonucleotides P9 and P10 (Table 1) with sequences complementary to these PBS are hybridized to form duplex enzyme recognition sequences (step 2). The partially duplex products are first digested with Nb.BtsI nicking endonuclease (step 3). This is followed by bead binding of biotinylated DNA to remove 3′ end digestion fragments (step 4). The desired ssDNA band is released from the beads by multiple rounds of on-bead BspQI digestion (step 5). One round of negative selection (Step 6) is done using beads partially pre-coated with oligonucleotide P7 and P8 (approx. 50% of the binding capacity) to remove partially digested DNA, excess of oligonucleotide P10 and any biotinylated products that may have failed to capture at previous steps. P9 oligonucleotide has two degenerate 3′ end nucleotides (5′—GCAGTGNN—3′) to improve digestion efficiency of nicking endonuclease

Figure 4b and 4c show the sequential removal of PBS. A 94–96 nucleotides long RNA library (lane A) was converted into a 107–109 nucleotides cDNAs (lane C) following the method described in figure 3a. The product of the IVT can be totally hydrolyzed with sodium hydroxide (lane B), demonstrating that RNA was made. The single strand library obtained from step 5 is shown on Figure 4b, lane E. The 49–51 mers library corresponds to the lowest and most intense band. There are two discrete bands above. The top one corresponds to fragments that failed to be cut by BspQI (89–91 nucleotides) while the middle band corresponds to fragments that failed to be cut by Nb.BtsI (67–69 nucleotides). When this sample is submitted to one round of negative selection (step 6), these two discrete bands disappear, as seen on Figure 4c, lane E. The single stranded nature of the cDNA and the released oligonucleotide library has been confirmed by a total degradation of these products when subjected to ssDNA-specific exonuclease I treatment, lane D and F respectively. Here, 100 pmol of biotinylated cDNA after PBS removal and affinity purification gives 50–60 pmol of PBS-free ssDNA. In contrast, the yield of PBS-free ssDNA achieved by PAGE purification is 30–35 pmol (data not shown). Thus for 7500 pmol biotinylated cDNA, the amount of PBS-free ssDNA is 3750 pmol by affinity selection (∼50% recovery) and 2625 pmol by gel purification (∼30% recovery).

### Determination of library coverage

**Figure 5:**
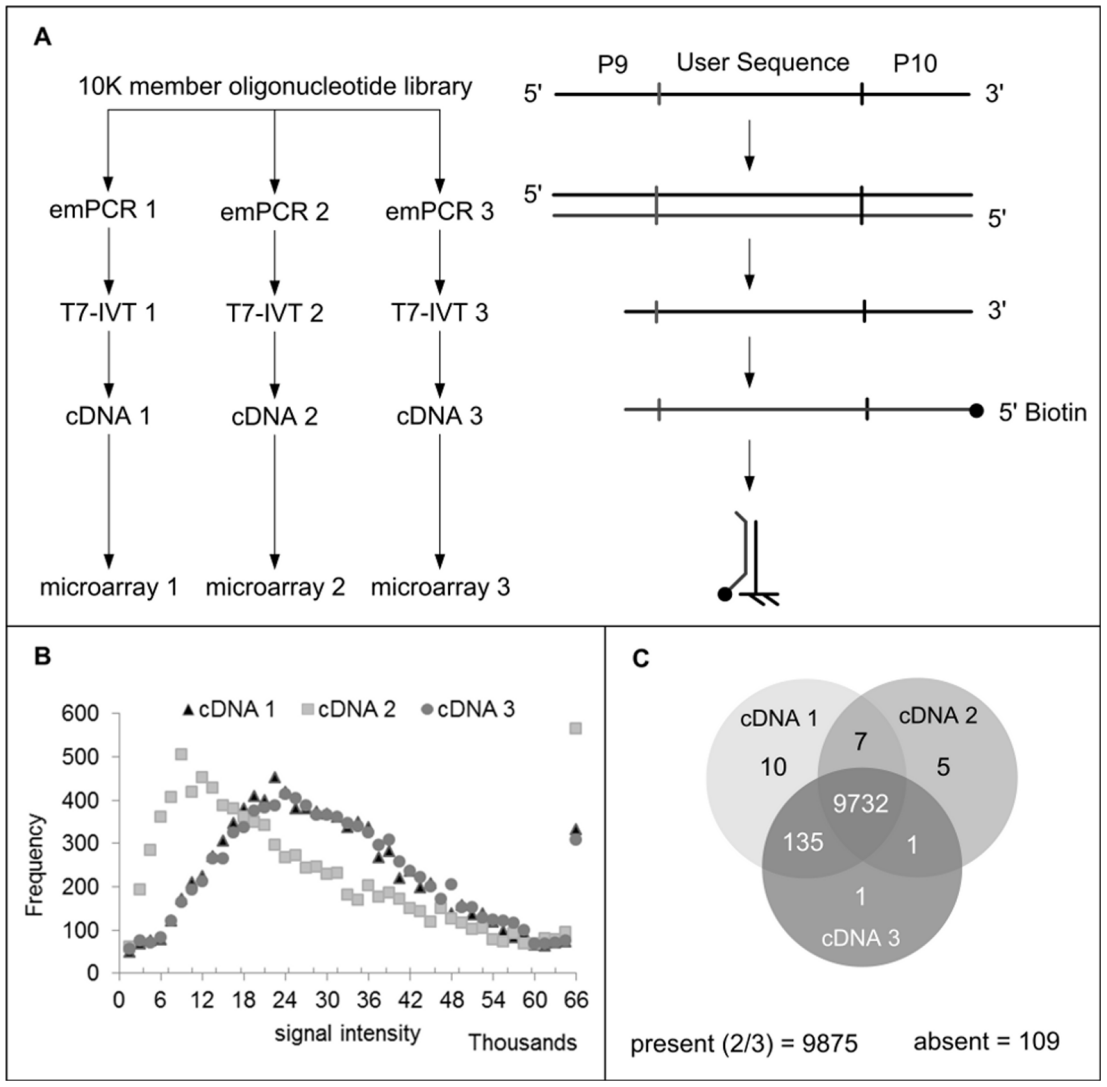
Determination of library coverage. (a) Experimental design. (b) Distribution of cDNA signal intensity. (c) Venn diagram – Present call per array.

The experimental design to study coverage of ssDNA libraries obtained by *in vitro* transcription and reverse transcription is depicted in fig 5a. Here, the 10,000 member library is amplified thrice independently by emulsion PCR. The resultant amplicons are subjected to IVT-RT method to generate biotinylated cDNAs (125–126 nt). The cDNA have 3 distinct regions: 5′-common sequence (68 nt)/46–47 nt variable region/common sequence (19 nt) -3′. The library coverage is determined by hybridization of variable region (46–47mer) to corresponding microarray.

Figure 5b shows the frequency distribution of the 10K cDNA library on the basis of signal intensity, while the number of cDNA oligos called ‘present’ (signal intensity >3,000 units) across the 3 independent emPCR-IVT-RT reactions is summarized as a Venn diagram (fig 5c). Here, greater than 95% cDNA are detected for each of the reaction. The pronounced shift of low intensity signal (<15,000 units) in sample 2, introduces a 1.35% coverage bias for commonly detected oligonucleotide between all samples (9732) and samples 1 and 3 only (9867). Finally, less than 1.5% oligonucleotides (105 of 10,000) are labeled as ‘absent’.

## Discussion

Massively parallel oligonucleotide synthesis on microarrays has the advantage to produce hundreds of thousand different sequences on a single planar substrate. However, one drawback of this technology is the limited spot size where the synthesis occurs, which is usually well below 100 microns diameters [41], [42]. This results in very small synthesis scale (femtomole). In order to produce workable amount of oligonucleotides, it is necessary and more economical to go through a molecular amplification procedure, PCR being the easiest one, but also comes with its own limitations. Indeed, PCR amplification requires the presence of primer binding sites at each end of the oligonucleotides and will produce a double stranded amplicon, both being undesirable for most oligonucleotide library applications. In the present work, we have compared three different approaches to remove primer binding sites and deliver libraries of single-stranded oligonucleotides, namely alkaline denaturation, exonucleolytic strand removal and in vitro transcription-reverse transcription.

While alkaline denaturation is appealing for its simplicity, we have highlighted several drawbacks. First, the primer binding sites are removed by using nicking enzymes to specifically cleave the PBS of the desired strand. There is a very limited repertory of nicking enzyme, making it quasi impossible to design an oligonucleotide library omitting these recognitions sites. Second, the mild alkaline denaturing conditions used to melt the double stranded DNA, break the biotin – streptavidin bond to a significant extend, leading to the contamination of the desired product with complementary strands. While these strands could be removed by a second binding to beads, there is a risk that they have re-hybridized to a complementary strand. One could propose to first heat denature the PCR amplicons and then perform the removal of unwanted biotinylated strands with magnetic beads. This would work for complex libraries but will undoubtedly fail for low complexity ones, such as libraries of point-mutations of the same oligonucleotide coding for short polypeptides. Finally, in our hands alkaline denaturation method with a biotin-streptavidin affinity selection necessitated PAGE purification to get desired size ssDNA resulting in very low yields and we do not recommend it.

For the generation of small amounts of oligonucleotide (up to 1 µg), exonucleolytic strand removal is the preferred approach. Lambda exonuclease degrades the phosphorylated strand with much greater affinity than the non-phosphorylated (hydroxylated) one. However, over-treatment will lead also to the degradation of the desired strand, hence one need to perfectly control the reaction conditions (DNA concentrations, units of lambda exonuclease, temperature and incubation time). It is possible to protect the desired strand by using either a biotinylated PCR primer or introducing 3 or more phosphorothioate bonds in the PCR primer corresponding to the 5′ end of the desired strand. The lambda exonuclease strand removal has relatively low yield, but its simplicity is appealing when only a limited amount of oligonucleotide library is needed.

When large amounts of oligonucleotides are requested, PCR amplification and exonucleolytic strand removal yields are not sufficient. PCR amplification can be followed by in vitro transcription using a phage RNA polymerase such as T7. It has several advantages. First, the T7 polymerase will copy each DNA template >1000 times [38], [43]. Second, it produces single-stranded RNA molecules, which display a different spectrum of sensitivity to enzymatic or chemical reagents for ease of removal on the unwanted complementary strand. Third, RNA molecules can be copied back into DNA molecules by reverse transcription. Here, we have demonstrated that a double-stranded PCR amplicon can be amplified up to 750- fold into a RNA library. The reverse transcription process generates the desired single-stranded product and the RNA template can be specifically degraded in alkaline conditions. However, T7 transcription adds 3 bases (GGG) to the 5′ end of the RNA due to the nature of the polymerase promoter and transcription start [44], [45]. Furthermore, the reverse transcription needs to be primed. Thus both ends of the cDNA need to be cleaned. We have demonstrated that this can be readily achieved by hybridizing complementary oligonucleotides to these ends and using restriction enzymes to cut the primers off the desired strand. Most restriction enzymes cannot cut a single-stranded template, thus one can have a particular restriction site in the oligonucleotide library without losing this particular oligonucleotide from the pool. As the procedure to remove PCR-primers and purification are the same for lambda exonuclease and IVT-RT method, we expect to get >450- fold more desired ssDNA when starting from the same amount of emulsion PCR product (Table S1 and S2).

The use of biotin moiety at 5′-terminus ssDNA and the need for only PBS regions to be duplexed facilitates the use of affinity purification methods to obtain desired ssDNA. This gives approximately 1.6- fold more recovery of desired ssDNA (no partial digests and smaller DNA fragments) than purification using denaturing polyacrylamide gels. Another advantage of affinity methods is that the oligonucleotides synthesized on microarray can be of different lengths. This is not possible when using gel purification methods. As a next step, we propose an oligonucleotide design that uses a single enzyme (BspQI) to remove both PBS at once in solution prior to capture on beads (Figure S1). This would eliminate the need to perform a negative selection round to remove incompletely digested fragments as well as removal of PBS on the beads.

The synthesis of ssDNA by IVT-RT method has a low oligonucleotide dropout percent (∼1.05%) from the pool as detected on microarray. Besides, reverse transcription failure, there are multiple possible causes for missing oligos from the pool, i) microarray synthesis failure, ii) dropout during emulsion PCR, and iii) lack of hybridization to detection probe (due to probe synthesis failure or oligo secondary structure and/or steric hindrance). Alternative conclusions cannot be ruled out. Ideally, a library of individually synthesized oligonucleotides would be pooled at equimolar concentration and subjected to PCR then IVT-RT amplification and used for normalization. This approach is too expensive to be practical. Another approach would be to perform deep sequencing of the original and the amplified library. However current NGS platforms also come with inherent biases and preferential sequence drop-out during sequencing is possible.

To summarize, applications requiring less than 1 µg of single stranded oligonucleotide libraries, we recommend using the exonucleolytic strand removal because of its simplicity. However, when larger amounts are requested, it becomes necessary to implement the in vitro transcription – reverse transcription method. It is important to note that the present reverse transcription method can also be followed by a second strand synthesis offering an appealing alternative to large scaling up of emulsion PCR amplification when double stranded libraries are considered. A major application of double-stranded library is cloning into a vector and in this case, the PBS are usually removed using restriction enzymes compatible with the targeted vector.

## Supporting Information

Figure S1 **Oligonucleotide design (BspQI restriction enzyme site at both ends).**
(DOC)

Table S1 **Empirical comparison of ssDNA yield between IVT-RT and lambda exonuclease method for minimum size reaction as recommended by kit manufacturer's (especially for IVT).**
(DOC)

Table S2 **Theoretical ssDNA yield with IVT-RT method from use of a single 100 µl emulsion PCR amplicons.**
(DOC)
