# Supplementary material for: Methods for the Preparation of Large Quantities of Complex Single-Stranded Oligonucleotide Libraries
Source: PLoS One. 2014 Apr 14;9(4):e94752. doi: 10.1371/journal.pone.0094752 (PMC3986247; doi:10.1371/journal.pone.0094752)
Supplement: Figure S1 — Oligonucleotide design (BspQI restriction enzyme site at both ends). (DOC) [file pone.0094752.s001.doc]

**Methods for the Preparation of Large Quantities of Single-Stranded Oligonucleotide Libraries**

**Yusuf E. Murgha^a^, Jean-Marie Rouillard^b,1^, Erdogan Gulari^b^**

**^a^Department of Biomedical Engineering, University of Michigan, Ann Arbor, MI**

**^b^Department of Chemical Engineering, University of Michigan, Ann Arbor, MI**

^1^To whom correspondence may be addressed. Mail: Jean-Marie Rouillard, Chemical Engineering, 2300 Hayward St., 3074 H.H. Dow Building, Ann Arbor, MI 48109-2136; Phone: (734) 763 4722; Email: [jmrouill@umich.edu](mailto:jmrouill@umich.edu)

**Figure S1. Oligonucleotide design (BspQI restriction enzyme site at both ends)**

5’/Bio/(dA)_30_------GCTCTTCN|NNN 5’-NGAAGAGC----3’/Bio/

3’------- CGAGAAGNNNN|-------------------------|NCTTCTCG------5’/Bio/
