# Supplementary material for: Methods for the Preparation of Large Quantities of Complex Single-Stranded Oligonucleotide Libraries
Source: PLoS One. 2014 Apr 14;9(4):e94752. doi: 10.1371/journal.pone.0094752 (PMC3986247; doi:10.1371/journal.pone.0094752)
Supplement: Table S1 — Empirical comparison of ssDNA yield between IVT-RT and lambda exonuclease method for minimum size reaction as recommended by kit manufacturer's (especially for IVT). (DOC) [file pone.0094752.s002.doc]

**Methods for the Preparation of Large Quantities of Single-Stranded Oligonucleotide Libraries**

**Yusuf E. Murgha^a^, Jean-Marie Rouillard^b,1^, Erdogan Gulari^b^**

**^a^Department of Biomedical Engineering, University of Michigan, Ann Arbor, MI**

**^b^Department of Chemical Engineering, University of Michigan, Ann Arbor, MI**

^1^To whom correspondence may be addressed. Mail: Jean-Marie Rouillard, Chemical Engineering, 2300 Hayward St., 3074 H.H. Dow Building, Ann Arbor, MI 48109-2136; Phone: (734) 763 4722; Email: [jmrouill@umich.edu](mailto:jmrouill@umich.edu)

**Table S1. Empirical comparison of ssDNA yield between IVT-RT and lambda exonuclease method for minimum size reaction as recommended by kit manufacturer’s (especially for IVT)**

| ***Reaction*** | ***Input*** | ***Output*** |
| --- | --- | --- |
| *Emulsion PCR* | 2.5 fmol per 100 μl | 25 pmol per 100 μl |
| **IVT-RT method** | | |
| *In vitro transcription* | 2 pmol per 20 μl | 1500 pmol per 20 μl |
| *Reverse transcription* | 1500 pmol | 750 pmol* |
| *PBS-free ssDNA (affinity isolation)* | 750 pmol | 375 pmol |
| **Lambda exonuclease method – constant emulsion PCR product concentration** | | |
| Lambda exonuclease | 25 pmol | 20 pmol |
| PBS-free ssDNA (affinity isolation) | 20 pmol | 10 pmol |

* The efficiency of reverse transcription reaction limits the final yield of ssDNA with the IVT-RT method

**Table S2. Theoretical ssDNA yield with IVT-RT method from use of a single 100 μl emulsion PCR amplicons**

| ***Reaction*** | ***Input*** | ***Output*** |
| --- | --- | --- |
| *Emulsion PCR* | 2.5 fmol | 25 pmol |
| **IVT-RT method** | | |
| *In vitro transcription* | 25 pmol | 18750 pmol |
| *Reverse transcription* | 18750 pmol | 9375 pmol |
| *PBS-free ssDNA (affinity isolation)* | 9375 pmol | 4687 pmol* |
| **Lambda exonuclease method** | | |
| Lambda exonuclease | 25 pmol | 20 pmol |
| PBS-free ssDNA (affinity isolation) | 20 pmol | 10 pmol* |

*IVT-RT method gives > 450-fold PBS-free ssDNA than lambda exonuclease method

**Figure S1. Oligonucleotide design (BspQI restriction enzyme site at both ends)**

5’/Bio/(dA)_30_------GCTCTTCN|NNN 5’-NGAAGAGC----3’/Bio/

3’------- CGAGAAGNNNN|-------------------------|NCTTCTCG------5’/Bio/
